# Supplementary material for: GFI1B and LSD1 repress myeloid traits during megakaryocyte differentiation
Source: Commun Biol. 2024 Mar 28;7:374. doi: 10.1038/s42003-024-06090-z (PMC10978956; doi:10.1038/s42003-024-06090-z)
Supplement: Supplementary file 2 — Supplementary Information [file 42003_2024_6090_MOESM2_ESM.pdf]

Supplementary Figures

Supplementary Figure 1

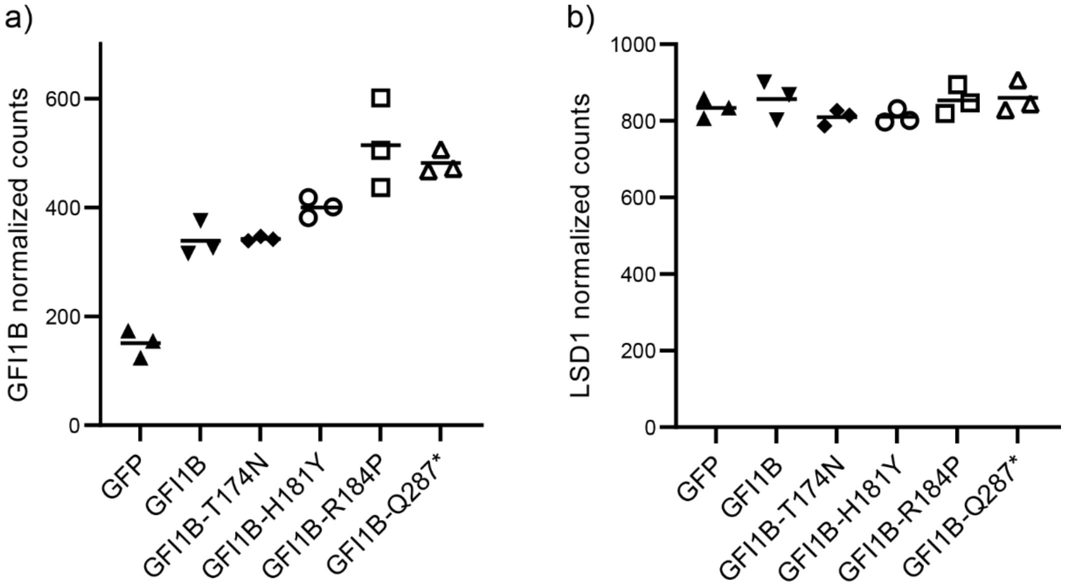

Supplementary Figure 1: GFI1B and LSD1 expression in MEG01 cells transduced with retroviral constructs containing GFP, wild type GFI1B, GFI1B^{T174N}, GFI1B^{H181Y}, GFI1B^{R184P}, or GFI1B^{Q287*}. a) Overexpression of GFI1B constructs induce a 2-3 fold upregulation of GFI1B compared to empty control (GFP). b) Retroviral overexpression of GFI1B constructs does not change LSD1 expression levels compared to empty control.

Supplementary Figure 2

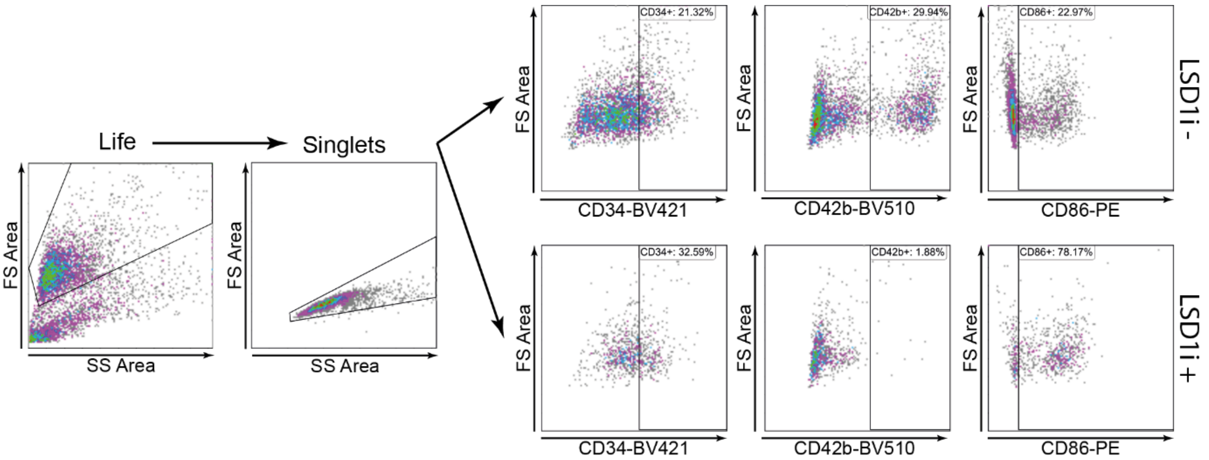

Supplementary Figure 2: Gating strategy for flow analysis. Life cells were gated out based on forward scatter area/Side scatter area. Doublets were removed using forward scatter area/side scatter area. Marker-positive cells were determined based on forward scatter area/marker area. Gate was set based on unstained samples. Plots shown here are representative of individual samples.

Supplementary Figure 3

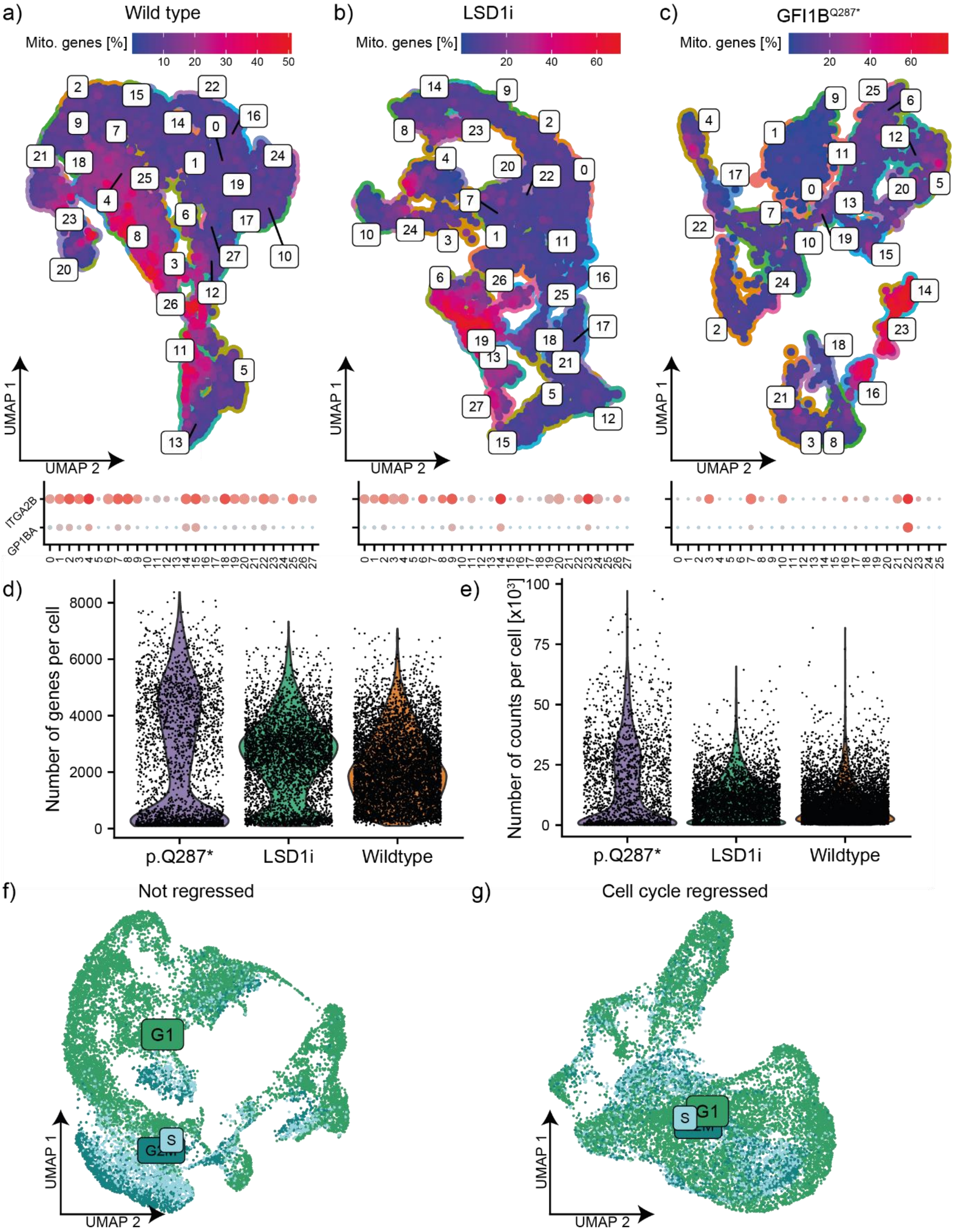

Supplementary Figure 3: Quality control and integration of scRNA samples derived from wild type, LSD1 inhibitor treated, and GFI1B^{Q287*} iPSCs produced a high quality scRNA dataset a-c) UMAP showing mitochondrial gene percentage for wild type (a), LSD1i (b) and GFI1B^{Q287*} (c) samples. Cluster 19 and 13 from the LSD1i sample and cluster 16, 23, and 14 from the GFI1B^{Q287*} sample were removed, because of their high mitochondrial gene percentage and upregulation of mitochondrial genes. d/e) The GFI1B^{Q287*} sample had a population of cells with more genes (d) and counts (e) per cell than other

cells from that sample. The gene and count distributions were comparable for the LSD1i and wild type samples. f) Integration of all samples revealed cell cycle to be a confounder in the analysis. g) All three samples were integrated and regressed for cell cycle, removing the confounding effect of cell cycle.

Supplementary Figure 4

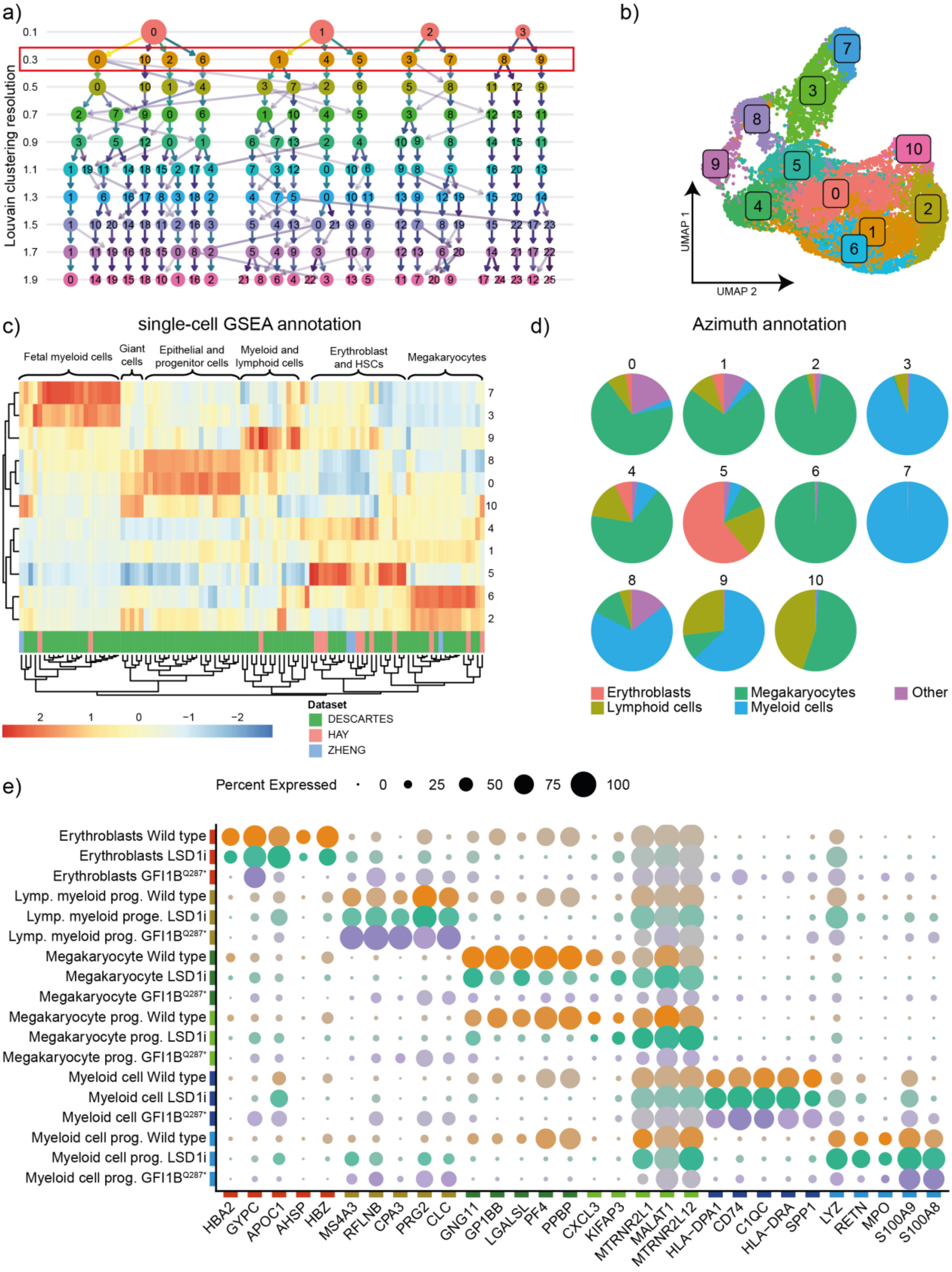

Supplementary Figure 4: Analysis of the scRNA dataset derived from wild type, LSD1 inhibitor treated, and GFI1B^{Q287*} iPSCs revealed six different cell types. a) Cells were clustered using the Louvain clustering algorithm. Clustree shows cluster distribution at different resolutions. Resolution 0.3 was chosen for further analysis, since all clusters appear to be stable from that point onward. b)

UMAP showed distinct clusters at resolution 0.3 c) All cells were analyzed using single-cell GSEA to determine enrichment of cell type gene sets. Clusters 2 and 6 showed enrichment for megakaryocyte gene sets and cluster 5 for erythroblast gene sets. Clusters 7 and 3 showed enrichment for myeloid gene sets and clusters 0 and 8 were associated to progenitor cell types. Cluster 9 was characterized by myeloid and lymphoid gene sets. d) Azimuth was used as a second cell type annotation tool. Clusters 3, 7, 8, and 9 were enriched for myeloid cells. Clusters 0, 1, 2, 4, 6 and 10 were enriched for megakaryocytes and cluster 5 was enriched for erythroblasts. Cluster 0 and 8 look similar using the scGSEA approach but show clear megakaryocyte and myeloid signatures, respectively, using Azimuth, highlighting the importance of using different annotation tools. Cluster 4 is characterized by a high erythroblast, HSCs, and megakaryocyte signature in the scGSEA but predominantly megakaryocyte using Azimuth. Therefore, we decided us to annotate them as megakaryocyte progenitors. e) Erythroblasts were characterized by hemoglobin-related genes. Megakaryocytes showed upregulation of canonical megakaryocyte genes, such as *GP1BB* and *PF4*. Myeloid cells were characterized by myeloid-related genes, such as *CD74* and *HLA*-genes.
