# Supplementary material for: GFI1B and LSD1 repress myeloid traits during megakaryocyte differentiation
Source: Commun Biol. 2024 Mar 28;7:374. doi: 10.1038/s42003-024-06090-z (PMC10978956; doi:10.1038/s42003-024-06090-z)
Supplement: Supplementary file 3 — Description of Additional Supplementary Files [file 42003_2024_6090_MOESM3_ESM.pdf]

Description of Additional Supplementary Files

File name: Supplementary Data 1
Description: Weighted Gene Co-Expression analysis yielded 12 modules with different sets of genes. The table contains the ensmbl identifiers and gene names for all genes that belong to a particular module.

File name: Supplementary Data 2
Description: Each module resulting from the Weighted Gene Co-Expression analysis was tested for enrichment of REACTOME pathways using g:Profiler. The resulting table was downloaded and contains enrichment of REACTOME pathways and an adjusted p-values for each module.

File name: Supplementary Data 3
Description: Megakaryocytes from the scRNA dataset were probed for differentially expressed genes by comparing GFI1B^{Q287*} and LSD1i inhibitor treated megakaryocytes with wild type. The resulting list of differentially expressed genes are shown. Genes that are upregulated in GFI1B^{Q287*} and LSD1 inhibitor treated megakaryocytes are highlighted with CommonDOWN. Genes that are upregulated in wild type megakaryocytes compared to GFI1B^{Q287*} and LSD1 inhibitor treated megakaryocytes are highlighted with CommonUP. The analysis showed that myeloid genes are commonly upregulated in GFI1B^{Q287*} and LSD1 inhibitor treated megakaryocytes.

File name: Supplementary Data 4
Description: SCENIC finds sets of genes that are commonly regulated by a transcription factors called a regulon. It outputs the activity score of every regulon within every single-cell. This table shows the activity for every regulon in all cells of the scRNA dataset.
