# Supplementary material for: GFI1B and LSD1 repress myeloid traits during megakaryocyte differentiation
Source: Commun Biol. 2024 Mar 28;7:374. doi: 10.1038/s42003-024-06090-z (PMC10978956; doi:10.1038/s42003-024-06090-z)
Supplement: Supplementary file 8 — Reporting Summary [file 42003_2024_6090_MOESM8_ESM.pdf]

nature portfolio

Corresponding author(s): Bert van der Reijden; Joost Martens

Last updated by author(s): Mar 6, 2024

Reporting Summary

Nature Portfolio wishes to improve the reproducibility of the work that we publish. This form provides structure for consistency and transparency in reporting. For further information on Nature Portfolio policies, see our Editorial Policies and the Editorial Policy Checklist.

Statistics

For all statistical analyses, confirm that the following items are present in the figure legend, table legend, main text, or Methods section.

n/a	Confirmed	
☐	☒	The exact sample size (*n*) for each experimental group/condition, given as a discrete number and unit of measurement
☐	☒	A statement on whether measurements were taken from distinct samples or whether the same sample was measured repeatedly
☒	☐	The statistical test(s) used AND whether they are one- or two-sided<br>*Only common tests should be described solely by name; describe more complex techniques in the Methods section.*
☒	☐	A description of all covariates tested
☒	☐	A description of any assumptions or corrections, such as tests of normality and adjustment for multiple comparisons
☒	☐	A full description of the statistical parameters including central tendency (e.g. means) or other basic estimates (e.g. regression coefficient) AND variation (e.g. standard deviation) or associated estimates of uncertainty (e.g. confidence intervals)
☒	☐	For null hypothesis testing, the test statistic (e.g. *F*, *t*, *r*) with confidence intervals, effect sizes, degrees of freedom and *P* value noted<br>*Give P values as exact values whenever suitable.*
☒	☐	For Bayesian analysis, information on the choice of priors and Markov chain Monte Carlo settings
☒	☐	For hierarchical and complex designs, identification of the appropriate level for tests and full reporting of outcomes
☒	☐	Estimates of effect sizes (e.g. Cohen's *d*, Pearson's *r*), indicating how they were calculated

Our web collection on statistics for biologists contains articles on many of the points above.

Software and code

Policy information about availability of computer code

Data collection	MSigDB website (Database v7.4)
Data analysis	R (v.4.1.1)<br>DESeq2 (v1.32)<br>Weighted Gene Co-expression Network analysis (v1.71)<br>Kaluza software v.2.1.2<br>Cellranger v.6.0.0<br>DropletUtils package (v1.10.3)<br>Seurat (v.4.0.1)<br>DoubletFinder (v.2.0.3)<br>Clustree (v.0.4.1)<br>escape (v.1.0.1)<br>pySCENIC (v0.11.2)<br>Arboreto (v0.1.6)<br>AUCell (v1.16.0)<br>MaxQuant (v1.6.6.6)

For manuscripts utilizing custom algorithms or software that are central to the research but not yet described in published literature, software must be made available to editors and reviewers. We strongly encourage code deposition in a community repository (e.g. GitHub). See the Nature Portfolio guidelines for submitting code & software for further information.

Data

Policy information about availability of data

All manuscripts must include a data availability statement. This statement should provide the following information, where applicable:

- Accession codes, unique identifiers, or web links for publicly available datasets
- A description of any restrictions on data availability
- For clinical datasets or third party data, please ensure that the statement adheres to our policy

All sequencing data have been submitted to GEO under accession numbers GSE244609 and GSE244756. The mass-spectrometry data has been submitted to PRIDE with accession PXD050401.

Research involving human participants, their data, or biological material

Policy information about studies with human participants or human data. See also policy information about sex, gender (identity/presentation), and sexual orientation and race, ethnicity and racism.

Reporting on sex and gender	NA
Reporting on race, ethnicity, or other socially relevant groupings	NA
Population characteristics	NA
Recruitment	NA
Ethics oversight	No human participants were included in this study. The study was conducted in accordance with the institutional guidelines and regulations from the Radboudumc Nijmegen (IRB number: CMO 2013/064).

Note that full information on the approval of the study protocol must also be provided in the manuscript.

Field-specific reporting

Please select the one below that is the best fit for your research. If you are not sure, read the appropriate sections before making your selection.

☒ Life sciences ☐ Behavioural & social sciences ☐ Ecological, evolutionary & environmental sciences

For a reference copy of the document with all sections, see nature.com/documents/nr-reporting-summary-flat.pdf

Life sciences study design

All studies must disclose on these points even when the disclosure is negative.

Sample size	No sample-size calculations were performed, since this is an in-vitro study.
Data exclusions	Single-cell sequencing data was filtered for low-quality cells prior to cell type and Single-Cell rEgulatory Network Inference and Clustering analysis and Low quality cells were defined as empty droplets that contain less then 100 UMIs, cells with a high percentage of mitochondrial genes and no marker gene expression, and cell-cell doublets.
Replication	Replication is indicated in the manuscript.
Randomization	NA
Blinding	NA

Reporting for specific materials, systems and methods

We require information from authors about some types of materials, experimental systems and methods used in many studies. Here, indicate whether each material, system or method listed is relevant to your study. If you are not sure if a list item applies to your research, read the appropriate section before selecting a response.

Materials & experimental systems

n/a	Involved in the study
☐	☒ Antibodies
☐	☒ Eukaryotic cell lines
☒	☐ Palaeontology and archaeology
☒	☐ Animals and other organisms
☒	☐ Clinical data
☒	☐ Dual use research of concern
☒	☐ Plants

Methods

n/a	Involved in the study
☒	☐ ChIP-seq
☐	☒ Flow cytometry
☒	☐ MRI-based neuroimaging

Antibodies

Antibodies used: CD34-561 Brilliant Violet 421 (Biolegend), CD86-IT2.2 PE (Biolegend), and CD42b-HIP1 Brilliant Violet 510 (Biolegend)

Validation: (BioLegend Cat# 343609, RRID:AB_11147951)
(BioLegend Cat# 305405, RRID:AB_314525)
(BioLegend Cat# 303933, RRID:AB_2716116)

Eukaryotic cell lines

Policy information about cell lines and Sex and Gender in Research

Cell line source(s): MEG01 were received from ATCC (ATCC-CRL-2021). K562 were received from ATCC (lot: 59300853). Induced pluripotent stem cells were obtained from Department of Hematopoiesis, Sanquin Research and Landsteiner Laboratory, Amsterdam, The Netherlands (Stem Cell Res 25, 34-37, doi:10.1016/j.scr.2017.10.008 (2017); Stem Cell Res 18, 26-28, doi:10.1016/j.scr.2016.12.004 (2017)).

Authentication: Cell lines were not authenticated

Mycoplasma contamination: Cell lines were tested regularly and negative for mycoplasma.

Commonly misidentified lines (See ICLAC register): No cell lines is reported to be commonly misidentified

Plants

Seed stocks: N/A

Novel plant genotypes: N/A

Authentication: N/A

Flow Cytometry

Plots

Confirm that:

☒ The axis labels state the marker and fluorochrome used (e.g. CD4-FITC).

☒ The axis scales are clearly visible. Include numbers along axes only for bottom left plot of group (a 'group' is an analysis of identical markers).

☒ All plots are contour plots with outliers or pseudocolor plots.

☒ A numerical value for number of cells or percentage (with statistics) is provided.

Methodology

Sample preparation: Cells were harvested and washed with PBS

Instrument	Beckman Coulter Gallios 10-color
Software	Kaluza 2.1.2
Cell population abundance	Around 70-90% of cells were 7-AAD negative (alive) after sorting.
Gating strategy	Life cells were gated using a FSC-Area/SSC-Area plot and doublets were removed based on FSC-Area/SSC-Area. Boundary between positive and negative populations were determined based on an unstained control sample.

☒ Tick this box to confirm that a figure exemplifying the gating strategy is provided in the Supplementary Information.
